# The High-Dietary Insulin Load Score Is Associated With Elevated Level of Fasting Blood Sugar in Iranian Adult Men: Results From Fasa PERSIAN Cohort Study

**DOI:** 10.1155/2024/6991072

**Published:** 2024-07-16

**Authors:** Seyede Hamide Rajaie, Sayyed Saeid Khayyatzadeh, Shiva Faghih, Yaser Mansoori, Mohammad Mehdi Naghizadeh, Mojtaba Farjam, Reza Homayounfar, Hassan Mozaffari-Khosravi

**Affiliations:** ^1^ Nutrition and Food Security Research Center Shahid Sadoughi University of Medical Sciences, Yazd, Iran; ^2^ Department of Nutrition School of Public Health Shahid Sadoughi University of Medical Sciences, Yazd, Iran; ^3^ Department of Community Nutrition Research Center School of Nutrition and Food Sciences Shiraz University of Medical Science, Shiraz, Iran; ^4^ Noncommunicable Diseases Research Center Diseases Research Center Fasa University of Medical Sciences, Fasa, Iran; ^5^ National Nutrition and Food Technology Research Institute Faculty of Nutrition Sciences and Food Technology Shahid Beheshti University of Medical Sciences, Tehran, Iran

**Keywords:** adults, dietary insulin index, dietary insulin load, hyperglycemia, metabolic syndrome

## Abstract

**Aim:** The potential of different foods to induce postprandial hyperinsulinemia may be involved in the development of metabolic syndrome (MetS). We aimed to evaluate the association between dietary insulin indices and MetS in a large population of adults in Iran.

**Methods:** A total of 6356 adults aged 35–70 years were included in the present cross-sectional study. A validated block-format 125-item semiquantitative food frequency questionnaire (FFQ) was used to obtain usual food intakes, and MetS was defined according to the International Diabetes Federation (IDF) and American Heart Association (AHA)/National Heart, Lung, and Blood Institute (NHLBI) criteria.

**Results:** MetS was prevalent in 13.8% of participants. Mean age of the study participants was 46.58 ± 8.82 years, and mean body mass index (BMI) was 25.02 ± 4.60 kg/m^2^. Mean dietary insulin index (DII) and dietary insulin load (DIL) were 63.15 ± 7.57 and 168.253 ± 52.09, respectively. In the crude model, men in the highest DIL quartile were more likely to have hyperglycemia than those in the lowest quartile (OR: 1.75, 95% CI: 1.12–2.73, *p* trend = 0.04). This association remained significant and was even stronger after adjusting for potential confounders in model I (OR: 3.64, 95% CI: 1.57–8.47, *p* trend = 0.005) and further adjustment for BMI in model II (OR: 3.61, 95% CI: 1.55–8.44, *p* trend = 0.006).

**Conclusions:** In healthy men, adherence to a high-DIL diet may be associated with a greater likelihood of having hyperglycemia. No statistically significant association was observed between insulin indices and the odds of having MetS.

## 1. Introduction

Metabolic syndrome (MetS) is becoming a worldwide health challenge [1], given that in most countries, about one-fifth of the adult population has this disorder [2]. The syndrome is an important risk factor for cardiovascular diseases, diabetes, and stroke [2–5] and is related to a 1.5-fold rise in the risk of all-cause mortality [6].

According to International Diabetes Federation (IDF) and American Heart Association (AHA)/National Heart, Lung, and Blood Institute (NHLBI) criteria [3], MetS is defined as the presence of at least three of the following components: (1) abdominal obesity (≥ 95 cm in men and women) [7], (2) high serum concentration of TG (≥ 150 mg/dL), (3) low serum concentration of high-density lipoprotein cholesterol (HDL-C) (< 50 mg/dL in women and < 40 mg/dL in men), (4) elevated blood pressure (systolic blood pressure [SBP] ≥ 130 mm Hg and/or diastolic blood pressure [DBP] ≥ 85 mm Hg), and (5) abnormal glucose homeostasis (FBG ≥ 100 mg/dL) [3]. Recent evidence indicates that dietary factors such as micronutrients and macronutrients, including carbohydrates, fiber, and fats, as well as usual dietary habits and patterns, are determining factors in the pathogenesis of insulin resistance [8, 9], which play a strong role in the pathogenesis of MetS and its components [10, 11].

Although the causes underlying the association between dietary factors and insulin resistance or MetS are not well understood, it was hypothesized that the ability of different foods to induce postprandial hyperinsulinemia might be involved in the development of insulin resistance, and therefore MetS [12].

Among dietary factors, carbohydrates are related to postprandial hyperglycemia and hyperinsulinemia [13]. However, postprandial blood insulin concentrations increase, independently of blood glucose concentrations, in response to other insulinotropic dietary factors such as fructose, certain amino acids, and some fatty acids [13, 14]. Thus, the glycemic index of foods, which is based solely on carbohydrate content, cannot indicate the relative insulin response to all dietary factors [15]. To systematically quantify the relative insulin response of all dietary insulinotropic factors, indices such as the dietary insulin index (DII) and dietary insulin load (DIL) have been suggested, which, in addition to the carbohydrate content of foods, also consider dietary proteins and fats and their interactions [15].

Few studies have assessed the associations between insulin indices and MetS [16] and its components [17, 18]. In a cross-sectional study that assessed the associations between insulin indices and MetS in 5954 Iranian adults (35–70 years), higher DIL and DII in women were associated with an increased odds of having MetS [16]. However, another cross-sectional study in 850 Iranian adults (20–59 years) found no associations between insulin indices and MetS nor between insulin indices and MetS components [17]. Moreover, a recent cross-sectional study in 203 Iranian adolescents with overweight or obesity found that insulin indices were positively associated with metabolic health status.

Given the inconsistent results of previous studies [16–18] and the lack of studies designed to examine the associations between dietary insulin indices and MetS and its components in a large sample of adults, we aimed to evaluate the association between dietary insulin indices and MetS in a large population of apparently healthy adults in Iran.

## 2. Materials and Methods

### 2.1. Study Population

This cross-sectional study was carried out with baseline data from the Fasa Prospective Epidemiological Research Studies in Iran (PERSIAN) cohort study. This ongoing cohort study is part of the PERSIAN Multicenter Cohort Study, which is following 10,138 adults 35–70 years old who live in the Sheshdeh region of Fasa (Sheshdeh town and its 24 surrounding villages), Fars Province, Iran. The study protocol of the Fasa PERSIAN Cohort Study was published in detail elsewhere [19].

As previously described [20], a multistage random cluster sampling method was used to select potential participants, and eligible participants were recruited after obtaining their written informed consent. Blood samples and information on general characteristics, demographic status, anthropometric indices, dietary intakes, and other lifestyle-related factors were collected from eligible participants. All data were collected with a pretested questionnaire in a face-to-face interview [19].

Data from 10,138 participants were used. Participants for whom data on dietary intake (*n* = 20) or outcomes of interest (*n* = 15) were missing and those who under- or over-reported their calorie intake (< 800 kcal/d or > 4200 kcal/d; *n* = 1278) were excluded. In addition, pregnant and lactating women and participants with a history of diseases such as cardiac ischemia, myocardial infarction, stroke, renal failure, or diabetes were also excluded due to the possibility of dietary modifications (*n* = 2429). Participants with a body mass index (BMI) of 40 or more were also excluded due to the possibility of underreporting dietary intake (*n* = 40). Therefore, 6356 persons were included in the final analysis.

### 2.2. Dietary Assessment

A validated block-format 125-item semiquantitative food frequency questionnaire (FFQ) [20, 21] was used to obtain usual food intakes during the previous year through face-to-face interviews with trained dietitians. The interviewers asked participants to report their average food intake frequencies daily, weekly, or monthly based on household measures. Then, portion sizes of food items were converted to grams. The US Department of Agriculture (USDA) nutrient database modified for Iranian foods was used to obtain the energy and nutrient contents of foods [22, 23].

### 2.3. DII and DIL Calculation

The food insulin index (FII) refers to the total insulin area under the curve over 2 h in response to consumption of a 1000-kJ portion of the test food divided by the area under the curve after ingestion of a 1000-kJ portion of the reference food. The insulin indexes of some food items were obtained from previous studies [15, 16, 24, 25], but indexes for other foods, including Iranian food items, were estimated as the FII of similar food items based on similarities between their energy, carbohydrate, protein, fat, and fiber content. The FII for three food items (coffee, tea, and salt) was assumed to be zero because their energy, carbohydrate, protein, and fat content are about zero.

The DIL for each participant was calculated by summing the insulin load of each food (FIL), which in turn was calculated as follows:

 $$\begin{array}{c} \text{FIL}=\text{insulin index of a given food}\times \text{energy content per }1\text{ g of that food}\times \text{amount }\left(\text{g}\right)\text{ of that food consumed per day}. \end{array}$$

The DII for each participant was calculated by dividing DIL by total energy intake [16].

### 2.4. Anthropometric and Blood Pressure Measurements

Weight was measured to the nearest 0.1 kg with a digital scale, with participants wearing light indoor clothing. Height (without shoes) was recorded to the nearest 0.5 cm with a tape measure. BMI was calculated as body weight (kg) divided by the square of height (m^2^). Waist circumference (WC) was measured to the nearest 0.1 cm with a plastic measuring tape after participants had removed their clothing, at the end of normal expiration without pressure on the body, at middistance between the iliac crest and the lowest rib [20, 26].

Participants were asked to rest for 10 min before their blood pressure was measured with a standard mercury sphygmomanometer. Two measurements from the right arm were recorded at a 15-min interval, and blood pressure was reported as the average of these two measurements.

### 2.5. Biochemical Assays

The samples were collected from participants after 12–14 h of overnight fasting. A volume of 25 mL of blood was drawn, centrifuged, and divided into aliquots labeled and stored at −70°C. In addition to the stored samples, a small amount of blood was used to measure concentrations of fasting blood glucose (FBG), triglycerides (TGs), and HDL-C. All measurements were obtained with an AutoAnalyzer system (Selectra E, Vitalab, Holliston, the Netherlands) and Pars Azmoon kits [20].

### 2.6. Assessment of Other Variables

A pretested questionnaire was used in a face-to-face interview to collect information on age (continuous), sex (male vs. female), education (university graduate vs. no university education), marital status (married vs. single or divorced), socioeconomic status (SES) (continuous), pregnancy or lactation status (yes/no), active smoking (currently smoking at least one cigarette a day) (yes/no), and previous diagnoses of any disease (yes/no).

Participants were asked about their physical activity during the previous year, and their physical activity level was expressed as metabolic equivalent hours per week (METs h/w) [27].

SES is reported here as wealth score index (WSI), estimated by multiple correspondence analysis (MCA) of the following variables: access to a freezer, access to a washing machine, access to a dishwasher, access to a computer, access to the internet, access to a motorcycle, access to a car (no access, access to a car costing < 11,000 US dollars, or access to a car costing > 11,000 US dollars), access to a vacuum cleaner, television type (no color television or regular color television vs. plasma color television), owning a mobile phone, owning a personal computer or laptop, and international trips in a lifetime (never, pilgrimage only, both pilgrimage or nonpilgrimage trips) [20].

### 2.7. Statistical Analysis

First, the normal distribution of continuous variables was assessed with the Kolmogorov–Smirnov test and histogram curves. We classified men and women into four categories of DIL and DII.

To assess differences in quantitative variables between categories of DIL and DII, one-way ANOVA, the Kruskal–Wallis H test, or the chi-squared test were used as appropriate. Dietary intakes across quartiles of DIL and DII were compared with ANCOVA to adjust for age and energy intake (kcal/d).

Binary logistic regression with adjusted models was used to calculate the odds ratios (ORs) and 95% confidence intervals (CIs) for MetS between categories of DIL and DII. In model 1, the effects of age, total energy intake (kcal/d), physical activity (continuous), home ownership, SES, education, marital status, and smoking status were adjusted for as potential confounders; further adjustment in model 2 was made for BMI. The first quartile of DIL or DII was considered the reference category in all models. The trend was determined with logistic regression models based on DIL and DII quartiles as ordinal variables.

All statistical analyses were done with SPSS version 21 (SPSS Inc, Chicago, IL, USA). Values of *p* were considered significant at < 0.05.

## 3. Results

After the eligibility criteria were applied, data for 6356 participants (mean age 46.58 ± 8.82 years, mean BMI 25.02 ± 4.60 kg/m^2^) were included in the present analysis. MetS was prevalent in 875 participants (13.8%). Mean DIL was 168.253 ± 52.091, and mean DII was 63.15 ± 7.57.

The general characteristics of participants across quartiles of DIL and DII are shown in Table 1. In men, those in the lowest DII quartile were more likely to have higher SES compared to those in the highest quartile (*p* < 0.001). Moreover, women in the highest DIL quartile were more likely to be university graduates (*p* = 0.001) than those in the lowest quartile. However, we observed no statistically significant differences for general and central obesity, physical activity, marital status, home ownership, smoking status, and having MetS among DII or DIL quartiles.

Age- and energy-standardized intakes of selected foods and nutrients across DIL and DII quartiles in men and women are shown in Tables 2 and 3. Men and women in the highest DIL quartile had higher intakes of cereals, meat, vegetables, fruits, and dairy products, as well as total energy, carbohydrates, protein, fat, total fat, dietary fiber, potassium, and sodium compared to the lowest quartile (*p* < 0.001 for all). In addition, both men and women in the highest DII quartile had higher intakes of whole grains, total energy, carbohydrates, protein, caffeine, and sodium and lower intakes of meats, fruits, vegetables, dairy products, total fat, dietary fiber, and potassium compared to those in the lowest quartile (*p* < 0.001 for all).

Multivariable-adjusted ORs for MetS and its components across DIL and DII quartiles are shown in Table 4. No statistically significant associations were observed between DIL or DII and the odds of having MetS in the crude or adjusted models. The results were similar when DIL and DII were adjusted for energy intake with the residual method (data not shown). However, in the crude model, men in the top DIL quartile were more likely to have hyperglycemia than those in the bottom quartile (OR: 1.75, 95% CI: 1.12–2.73, *p* trend = 0.04). This association remained significant and was even stronger after adjusting for age, total energy intake (kcal/d), physical activity (continuous), home ownership, SES, education, marital status, and smoking status in model I (OR: 3.64, 95% CI: 1.57–8.47, *p* trend = 0.005), and after further adjustment for BMI in model II (OR: 3.61, 95% CI: 1.55–8.44, *p* trend = 0.006).

In addition, in women, a marginally significant (*p* < 0.1) positive trend in the OR was observed for DIL with hypertension (model II) and with hypertriglyceridemia (crude model and model II). However, these associations are not clinically important.

## 4. Discussion

In this cross-sectional study of apparently healthy adults, no statistically significant associations were observed between insulin indices (DII and DIL) and having MetS. However, analysis with multivariate-adjusted models showed that in men, adherence to a diet with a high DIL was associated with a greater likelihood of having hyperglycemia.

In line with our findings, a recent cross-sectional survey on 850 Iranian adults showed that insulin indices (DII and DIL) were not significantly associated with MetS [17]. However, two other Iranian studies [16, 18] contradict these findings. Sadeghi et al. [16] showed that DIL and DII (in women) were positively associated with the odds of having MetS in apparently healthy adults. Moreover, Hajhashemy et al. [18] found that DIL and DII were positively associated with metabolic health status in 203 adolescents with overweight or obesity.

The lower mean BMI (approximately 25.07 kg/m^2^) in our participants compared to other studies [16, 18] might help to explain these inconsistent results, because some studies found that the associations between DIL and DII and health outcomes were strongest in overweight and obese individuals [12, 18]. Obesity is accompanied by some degree of insulin resistance and compensatory hyperinsulinemia [12, 28, 29]. Another explanation for this discrepancy may be masking of the effects of insulinotropic dietary factors by the high proportions of anti-MetS foods such as vegetables and whole grains consumed by our participants, given that these amounts were much higher than in other studies [16, 18]. Furthermore, other potential factors that may contribute to discrepancies among studies are differences in the criteria used to define MetS, other dietary factors (such as meal frequency and food combinations) that are not considered in DIL or DII calculations, and the type and number of confounding factors used for adjustment.

The present results show a significant positive correlation between DIL and the odds of having hyperglycemia in men, which is in line with other studies of the Iranian population [30–32]. In a cross-sectional study by Mozaffari et al. [32] of 357 older men, DIL was positively associated with FBS levels. Moreover, Mirmiran et al. [30] found a borderline direct association between DII and the risk of insulin resistance. In addition, DIL was positively associated with the risk of insulin resistance after 3 years in healthy adults. Teymoori et al. [31], who conducted a study of 1149 adults aged 30 years or older, reported that diets with high DII or DIL were positively associated with the incidence of diabetes. However, the present findings contrast with other studies [12, 17, 33] that used different DIL calculation methods [12], lower sample sizes [12, 17, 33], and different study populations [33].

Although the mechanisms underlying the associations between insulin indices and MetS and its components are unknown, it has been proposed that long-term adherence to a high-DIL or high-DII diet may decrease insulin sensitivity, diminish lipolysis, and also stimulate body fat development. These diets may also stimulate insulin and insulin growth factor-1 (IGF-1) secretion and subsequently preadipocyte proliferation, which may cause body fat formation [34]. In addition, foods with a high insulin index are rapidly digested, absorbed, and transformed into glucose, which leads to a rapid increase in blood glucose and insulin values and subsequently a rapid decrease in blood glucose flow [35]. The rapid reduction in blood glucose can decrease satiety and therefore contribute to excessive calorie intake, which is consequently associated with an increased risk of obesity [35, 36], and with MetS [37].

It is rather unexpected that women are less likely than men to develop insulin resistance and hyperglycemia given that they have lower skeletal muscle mass, higher circulation nonesterified fatty acid (NEFA) levels, myocyte lipid content, and fat mass. Men might have more insulin resistance than women due to their higher levels of visceral and hepatic adipose tissue, lower levels of adiponectin, and the absence of any protective effects of estrogen [38]. According to experimental studies, estrogen protects women from NEFA-induced insulin resistance and makes them more resistant to lipotoxicity, particularly in skeletal muscles [39]. In detail, estrogens activate the estrogen receptor *α* (ERɑ) pathway, which protects against insulin resistance and retains mitochondrial function in insulin-sensitive tissues [40]. For instance, in myocyte ERɑ deleted mice, muscle-associated oxidative metabolism was changed, and hyperglycemia ensued [41].

The present study has several strengths: (1) its large sample size of adults, (2) access to different blood pressure measurements and biochemical test results, (3) consideration of several potential confounders, and (4) use of a validated and reliable FFQ to determine dietary intakes.

We are also aware of some study limitations. (1) The cross-sectional design cannot address causality or the direction of the relationships. (2) Iranian foods were not considered in our FII calculations. Thus, slight differences may occur between the FII of the test foods in reference sources and the foods consumed by our participants. (3) Although we used validated assessment and measurement tools, misclassification or measurement error is inevitable. (4) Insulin scores are limited in that they are designed to assess total amounts of insulinogenic food intake and are not intended to measure other factors affecting the insulin response, for example, meal frequency and combinations of foods. Another limitation of insulin scores (DII and DIL) is that the FII values were obtained from a sample of lean university students [24], so these scores are likely to differ in other study populations such as older and heavier individuals.

## 5. Conclusions

In conclusion, our findings indicate that adherence to a high-DIL diet may be associated with a greater likelihood of having hyperglycemia in apparently healthy men. No statistically significant association was observed between DIL or DII and the odds of having MetS. Further studies, especially those with a prospective design, are required to shed light on the role of adhering to a diet with high insulin indices on the development of MetS.

## Figures and Tables

**Table 1:** Characteristics of the participants across quartiles of DII and DIL.

**Variable** ^a^	**Quartiles of DII**				**p** **value** ^b^	**Quartiles of DIL**				**p** **value** ^b^
	**1**	**2**	**3**	**4**		**1**	**2**	**3**	**4**	
*Men*						*Men*				
*n*	735	794	838	822		679	776	853	881	
Age (y)	47 ± 9	47 ± 9	47 ± 9	48 ± 9	0.48	48 ± 9	47 ± 9	47 ± 9	47 ± 9	0.34
General obesity (%)^c^	60 (8.2)	60 (7.6)	62 (7.4)	59 (7.2)	0.90	58 (8.5)	55 (7.1)	58 (6.8)	70 (7.9)	0.55
Abdominal obesity (%)^c^	188 (25.6)	196 (24.7)	243 (29)	211 (25.7)	0.20	186 (27.4)	195 (25.1)	215 (25.2)	242 (27.5)	0.54
Physical activity (MET-h/w)	46.29 ± 14.95	45.46 ± 14.28	45.82 ± 14.26	45.70 ± 14.29	0.72	45.90 ± 14.16	45.32 ± 14.40	46.07 ± 15.03	45.91 ± 14.09	0.74
Education (university graduate %)	38 (5.2)	32 (4)	27 (3.2)	30 (3.6)	0.23	34 (5)	32 (4.1)	30 (3.5)	31 (3.5)	0.41
Marital status (married %)	705 (95.9)	767 (96.6)	812 (96.9)	807 (98.2)	0.06	659 (97.1)	742 (95.6)	833 (97.7)	857 (97.3)	0.09
Home ownership (owner %)	670 (91.2)	727 (91.6)	770 (91.9)	749 (91.1)	0.93	607 (89.4)	714 (92)	780 (91.4)	815 (92.5)	0.15
Socioeconomic status^d^	−0.07 (−0.95 to 2.61)	−0.25 (−1.11 to 1.96)	−0.31 (−1.15 to 1.99)	−0.40 (−1.19 to 1.30)	< 0.001	−0.18 (−1.07 to 2.40)	−0.20 (−1.05 to 2.12)	−0.34 (−1.17 to 1.85)	−0.38 (−1.16 to 1.51)	0.10
Active smoker (%)	316 (43)	361 (45.5)	343 (40.9)	325 (39.5)	0.08	296 (43.6)	349 (45)	360 (42.2)	340 (38.6)	0.053
MetS (%)^e^	86 (11.7)	101 (12.7)	105 (12.5)	91 (11.1)	0.71	81 (11.9)	89 (11.5)	98 (11.5)	115 (13.1)	0.71
*Women*						*Women*				
*n*	854	795	751	767		910	813	736	708	
Age (y)	46 ± 9	46 ± 9	46 ± 9	46 ± 8	0.64	46 ± 9	46 ± 8	46 ± 8	46 ± 8	0.38
General obesity (%)^c^	190 (22.2)	175 (22)	160 (21.3)	150 (19.6)	0.55	211 (23.2)	179 (22)	147 (20)	138 (19.5)	0.22
Abdominal obesity (%)^c^	404 (47.3)	384 (48.3)	368 (49)	389 (50.7)	0.57	445 (48.9)	393 (48.3)	357 (48.5)	350 (49.4)	0.97
Physical activity (MET-h/w)	38.75 ± 7.31	38.42 ± 6.96	38.59 ± 6.16	38.95 ± 6.65	0.46	38.61 ± 6.94	38.64 ± 6.90	38.29 ± 6.40	39.20 ± 6.91	0.08
Education (university graduate %)	17 (2)	18 (2.3)	5 (0.7)	12 (1.6)	0.07	26 (2.9)	7 (0.9)	14 (1.9)	5 (0.7)	0.001
Marital status (married %)	691 (80.9)	672 (84.5)	612 (81.5)	650 (84.7)	0.08	737 (81)	687 (84.5)	613 (83.3)	588 (83.1)	0.27
Home ownership (owner %)	760 (89)	721 (90.7)	694 (92.4)	696 (90.7)	0.13	819 (90)	725 (89..2)	675 (91.7)	652 (92.1)	0.15
Socioeconomic status^d^	−0.74 (−1.55 to 0.49)	−0.74 (−1.53 to 0.48)	−0.72 (−1.66 to 0.54)	−0.73 (−1.57 to 0.45)	0.51	−0.65 (−1.49 to 0.58)	−0.73 (−1.57 to 0.48)	−0.66 (−1.49 to 0.55)	−0.98 (−1.76 to 0.21)	0.01
Active smoker (%)	9 (1.1)	13 (1.6)	14 (1.9)	19 (2.5)	0.17	14 (1.5)	15 (1.8)	11 (1.5)	15 (2.1)	0.77
MetS (%)^e^	136 (15.9)	126 (15.8)	107 (14.2)	123 (16)	0.74	134 (14.7)	125 (15.4)	119 (16.2)	114 (16.1)	0.83

Abbreviations: DII = dietary insulin index, DIL = dietary insulin load.

^a^Data are presented as the mean ± standard deviation or absolute number (percentage); socioeconomic status is presented as the median (25th–75th percentile).

^b^Obtained from one-way ANOVA, Kruskal–Wallis H test, or chi-squared test, as appropriate.

^c^General obesity was defined as BMI ≥ 30 kg/m^2^, and central obesity was defined according to the specific definition developed for the Iranian population [7]: waist circumference ≥ 95 cm in men and women.

^d^Socioeconomic status is presented as wealth score index (WSI).

^e^MetS (metabolic syndrome) is defined according to the International Diabetes Federation (IDF) and American Heart Association (AHA)/National Heart, Lung, and Blood Institute (NHLBI) criteria [3].

**Table 2:** Age- and energy-standardized dietary intakes of the participants across quintiles of DIL scores.

	**Men**				**p** **value** ^a^	**Women**				**p** **value** ^a^
	**Quartiles of DIL**					**Quartiles of DIL**				
	**1**	**2**	**3**	**4**		**1**	**2**	**3**	**4**	
Range	≤ 129,604.34	129,637.65, 163,549.38	163,582, 204,149.01	≥ 204194	—	≤ 129634.85	129,645.76, 163,572.89	163,631.24, 204,054.49	≥ 204,162.14	—
*n*	679	776	853	881	—	910	813	736	708	—
Food groups (g/d)										
Cereals	389.91 ± 4.59	529.96 ± 4.56	647.02 ± 5.04	850.22 ± 6.57	< 0.001	372.09 ± 3.74	532.03 ± 4.35	653.08 ± 5.40	850.48 ± 7.41	< 0.001
Meats	64.25 ± 1.55	82.78 ± 1.63	96.94 ± 1.94	103.02 ± 1.97	< 0.001	57.00 ± 1.22	75.36 ± 1.59	83.04 ± 1.85	92.82 ± 2.18	< 0.001
Dairy products	151.05 ± 4.42	187.76 ± 4.82	215.41 ± 5.66	221.43 ± 5.65	< 0.001	165.18 ± 4.38	197.85 ± 5.16	217.09 ± 6.23	232.09 ± 6.67	< 0.001
Fruits	246.17 ± 6.56	343.53 ± 8.61	387.87 ± 9.57	428.12 ± 10.51	< 0.001	268.07 ± 6.60	345.48 ± 8.60	408.57 ± 11.37	414.88 ± 11.52	< 0.001
Vegetables	393.38 ± 8.32	447.05 ± 8.68	516.90 ± 9.23	565.19 ± 9.59	< 0.001	416.36 ± 7.32	478.24 ± 8.70	532.20 ± 10.27	605.01 ± 13.77	< 0.001
Nutrients										
Energy (kcal/d)	1812.42 ± 13.73	2405.03 ± 11.48	2898.15 ± 11.74	3542.55 ± 12.38	< 0.001	1768.41 ± 12.03	2411.41 ± 10.67	2886.05 ± 12.79	3553.21 ± 13.98	< 0.001
Carbohydrates (g/d)	292.42 ± 2.20	396.75 ± 1.80	481.29 ± 1.86	603.65 ± 2.32	< 0.001	287.23 ± 1.96	395.47 ± 1.66	483.02 ± 2.19	603.29 ± 2.61	< 0.001
Protein (g/d)	58.25 ± .57	77.49 ± 0.53	94.00 ± 0.63	114.63 ± 0.66	< 0.001	56.61 ± 0.49	77.43 ± 0.51	91.96 ± 0.61	114.64 ± 0.73	< 0.001
Fat (g/d)	49.12 ± .67	60.83 ± 0.65	71.19 ± 0.75	79.27 ± 0.65	< 0.001	47.57 ± 0.57	62.27 ± 0.69	70.25 ± 0.77	80.69 ± 0.75	< 0.001
Fiber (g/d)	19.78 ± .25	25.79 ± 0.26	30.59 ± 0.28	36.69 ± 0.30	< 0.001	20.36 ± 0.23	26.38 ± 0.25	31.46 ± 0.33	37.36 ± 0.36	< 0.001
Caffeine (mg/d)	132.18 ± 4.04	162.00 ± 4.44	195.51 ± 5.52	235.61 ± 6.42	< 0.001	124.22 ± 3.18	147.56 ± 3.72	182.78 ± 5.98	210.93 ± 6.99	< 0.001
Potassium (mg/d)	2699.27 ± 33.14	3378.24 ± 35.38	3935.83 ± 38.49	4445.44 ± 38.93	< 0.001	2744.84 ± 29.39	3433.56 ± 34.10	3963.52 ± 43.97	4473.16 ± 48.53	< 0.001
Sodium (mg/d)	3208.63 ± 40.37	3883.53 ± 36.97	4594.11 ± 41.14	5580.95 ± 41.98	< 0.001	3194.11 ± 35.10	4028.56 ± 37.96	4720.43 ± 42.01	5809.51 ± 60.15	< 0.001

*Note:* Data are presented as the mean ± standard error.

Abbreviation: DII = dietary insulin load.

^a^Calculated using multivariate ANCOVA. All variables except energy were adjusted for both energy intake and age. Energy was adjusted for age.

**Table 3:** Age- and energy-standardized dietary intakes of the participants across quintiles of DII scores.

	**Men**				**p** **value** ^a^	**Women**				**p** **value** ^a^
	**Quartiles of DII**					**Quartiles of DII**				
	**1**	**2**	**3**	**4**		**1**	**2**	**3**	**4**	
Range	≤ 58.24	58.24, 63.29	63.32, 68.14	≥ 68.15	—	≤ 58.23	58.25, 63.32	63.32, 68.14	≥ 68.15	—
*n*	735	794	838	822	—	854	795	751	767	—
Food groups (g/d)										
Cereals	522.59 ± 7.27	590.27 ± 7.02	656.38 ± 7.57	698.45 ± 8.61	< 0.001	488.48 ± 6.35	558.24 ± 7.37	623.17 ± 7.80	684.46 ± 9.13	< 0.001
Meats	104.69 ± 2.12	96.26 ± 1.88	85.82 ± 1.68	68.15 ± 1.69	< 0.001	91.22 ± 1.86	81.31 ± 1.74	71.29 ± 1.63	57.22 ± 1.48	< 0.001
Dairy products	244.69 ± 6.62	208.74 ± 5.20	189.61 ± 5.01	149.16 ± 4.08	< 0.001	244.52 ± 6.05	214.99 ± 5.83	188.31 ± 5.12	148.76 ± 4.50	< 0.001
Fruits	443.29 ± 10.89	378.97 ± 9.23	351.93 ± 9.46	267.79 ± 7.45	< 0.001	441.26 ± 10.38	392.90 ± 10.40	316.80 ± 8.13	250.53 ± 7.38	< 0.001
Vegetables	514.55 ± 9.70	488.60 ± 9.43	469.90 ± 8.57	478.04 ± 9.78	< 0.001	520.31 ± 9.22	515.90 ± 9.48	484.90 ± 9.76	481.24 ± 12.25	< 0.001
Nutrients										
Energy (kcal/d)	2688.82 ± 26.20	2676.16 ± 24.89	2753.17 ± 25.28	2775.82 ± 25.25	0.021	2537.57 ± 24.40	2559.97 ± 25.91	2613.24 ± 26.64	2665.89 ± 28.51	0.002
Carbohydrates (g/d)	423.29 ± 4.53	438.39 ± 4.21	465.19 ± 4.40	486.33 ± 4.53	< 0.001	401.41 ± 4.26	421.10 ± 4.44	439.94 ± 4.55	466.19 ± 5.00	< 0.001
Protein (g/d)	86.49 ± 1.00	87.62 ± 0.93	89.33 ± 0.91	88.64 ± 0.96	< 0.001	80.84 ± 0.89	82.67 ± 0.93	84.33 ± 0.96	85.04 ± 1.02	< 0.001
Fat (g/d)	78.20 ± 0.94	68.28 ± 0.74	63.51 ± 0.68	56.21 ± 0.61	< 0.001	73.70 ± 0.87	65.61 ± 0.78	61.24 ± 0.73	54.32 ± 0.68	< 0.001
Fiber (g/d)	29.44 ± 0.39	28.62 ± 0.35	28.78 ± 0.34	28.44 ± 0.33	< 0.001	28.98 ± 0.39	28.71 ± 0.37	27.66 ± 0.33	27.69 ± 0.35	< 0.001
Caffeine (mg/d)	151.90 ± 4.86	167.10 ± 4.39	191.87 ± 5.54	224.68 ± 6.59	< 0.001	143.16 ± 4.54	150.33 ± 3.84	165.65 ± 5.14	196.47 ± 6.43	< 0.001
Potassium (mg/d)	4011.05 ± 48.98	3714.32 ± 42.71	3601.83 ± 41.04	3421.38 ± 39.27	< 0.001	3887.66 ± 46.29	3705.38 ± 44.61	3466.47 ± 40.94	3265.02 ± 43.11	< 0.001
Sodium (mg/d)	4125.98 ± 51.21	4281.72 ± 49.03	4495.07 ± 49.86	4657.82 ± 51.64	< 0.001	4053.25 ± 48.45	4258.48 ± 52.39	4481.12 ± 61.61	4637.47 ± 56.97	< 0.001

*Note:* Data are presented as the mean ± standard error.

Abbreviation: DII = dietary insulin index.

^a^Calculated using multivariate ANCOVA. All variables except energy were adjusted for both energy intake and age. Energy was adjusted for age.

**Table 4:** Odds ratios and 95% confidence intervals for prevalence of metabolic syndrome and its components across quartiles of DII and DIL.

**Models**	**Quartiles of DII**				**p** **trend** ^a^	**Quartiles of DIL**				**p** **trend** ^a^
	**1**	**2**	**3**	**4**		**1**	**2**	**3**	4	
		*Men*					*Men*			
Range	≤ 58.24	58.24, 63.29	63.32, 68.14	≥ 68.15		≤ 129,604.34	129,637.65, 163,549.38	163,582, 204,149.01	≥ 204194	
*n*	735	794	838	822		679	776	853	881	
Metabolic syndrome										
Crude	1	1.10 (0.80–1.49)	1.08 (0.79–1.46)	0.93 (0.68–1.28)	0.71	1	0.95 (0.69–1.31)	0.95 (0.70–1.31)	1.10 (0.81–1.50)	0.71
Model ɪ^b^	1	1.11 (0.81–1.52)	1.05 (0.77–1.44)	0.92 (0.67–1.27)	0.67	1	0.98 (0.67–1.42)	0.98 (0.61–1.57)	1.13 (0.60–2.10)	0.87
Model ɪɪ^c^	1	0.98 (0.73–1.30)	0.84 (0.62–1.13)	1.02 (0.77–1.36)	0.59	1	1.008 (0.66–1.52)	1.05 (0.62–1.76)	1.15 (0.58–2.30)	0.65
Central obesity^d^										
Crude	1	0.95 (0.75–1.20)	1.18 (0.95–1.48)	1.005 (0.80–1.26)	0.20	1	0.89 (0.70–1.12)	0.89 (0.71–1.12)	1.004 (0.80–1.25)	0.54
Model ɪ	1	0.98 (0.77–1.24)	1.19 (0.95–1.50)	1.02 (0.80–1.29)	0.28	1	0.94 (0.71–1.25)	0.96 (0.68–1.37)	1.11 (0.69–1.77)	0.66
Model ɪɪ	1	0.94 (0.66–1.36)	1.28 (0.90–1.82)	0.91 (0.63–1.30)	0.19	1	0.99 (0.65–1.51)	1.05 (0.62–1.79)	1.01 (0.49–2.07)	0.98
Hypertension^e^										
Crude	1	0.90 (0.69–1.18)	0.82 (0.62–1.07)	0.81 (0.61–1.06)	0.39	1	0.91 (0.68–1.21)	1.01 (0.77–1.33)	0.98 (0.75–1.29)	0.86
Model ɪ	1	0.90 (0.68–1.18)	0.79 (0.60–1.05)	0.77 (0.58–1.01)	0.23	1	0.79 (0.56–1.11)	0.76 (0.50–1.15)	0.58 (0.33–1.02)	0.29
Model ɪɪ	1	0.90 (0.68–1.20)	0.77 (0.58–1.02)	0.75 (0.57–1.008)	0.17	1	0.78 (0.55–1.10)	0.75 (0.49–1.15)	0.56 (0.31–0.99)	0.21
Low HDL-C^f^										
Crude	1	0.99 (0.80–1.23)	0.98 (0.79–1.21)	0.97 (0.79–1.20)	0.99	1	0.93 (0.75–1.16)	0.96 (0.78–1.19)	0.92 (0.74–1.14)	0.88
Model ɪ	1	0.99 (0.79–1.22)	1.00 (0.80–1.23)	0.99 (0.80–1.23)	1.00	1	0.96 (0.75–1.24)	1.06 (0.77–1.46)	1.09 (0.71–1.68)	0.84
Model ɪɪ	1	1.001 (0.80–1.24)	1.001 (0.80–1.24)	0.99 (0.80–1.24)	1.00	1	0.96 (0.74–1.25)	1.08 (0.78–1.49)	1.09 (0.70–1.68)	0.82
Hyperglycemia^g^										
Crude	1	1.22 (0.81–1.84)	1.19 (0.79–1.79)	1.17 (0.78–1.77)	0.78	1	1.84 (1.17–2.89)	1.52 (0.96–2.39)	1.75 (1.12–2.73)	0.04
Model ɪ	1	1.22 (0.81–1.86)	1.18 (0.78–1.79)	1.15 (0.76–1.75)	0.79	1	2.52 (1.49–4.24)	2.49 (1.30–4.76)	3.64 (1.57–8.47)	0.005
Model ɪɪ	1	1.24 (0.81–1.88)	1.17 (0.77–1.77)	1.15 (0.75–1.75)	0.78	1	2.53 (1.50–4.27)	2.52 (1.31–4.83)	3.61 (1.55–8.44)	0.006
Hypertriglyceridemia^h^										
Crude	1	0.96 (0.77–1.20)	0.94 (0.75–1.17)	0.90 (0.72–1.12)	0.83	1	0.98 (0.78–1.24)	0.93 (0.74–1.16)	1.06 (0.85–1.32)	0.68
Model ɪ	1	0.98 (0.78–1.23)	0.94 (0.75–1.18)	0.91 (0.73–1.14)	0.86	1	0.99 (0.76–1.29)	0.95 (0.68–1.32)	1.10 (0.70–1.72)	0.68
Model ɪɪ	1	1.002 (0.79–1.27)	0.94 (0.74–1.18)	0.90 (0.71–1.14)	0.79	1	1.006 (0.76–1.33)	0.96 (0.68–1.37)	1.09 (0.68–1.74)	0.84
		*Women*					*Women*			
Range	≤58.23	58.25, 63.32	63.32, 68.14	≥68.15		≤129634.85	129645.76, 163572.89	163631.24, 204054.49	≥204162.14	
*n*	854	795	751	767		910	813	736	708	
Metabolic syndrome										
Crude	1	0.99 (0.76–1.29)	0.87 (0.66–1.15)	1.00 (0.77–1.31)	0.74	1	1.05 (0.80–1.37)	1.11 (0.85–1.46)	1.11 (0.84–1.45)	0.83
Model ɪ	1	1.11 (0.81–1.52)	1.05 (0.77–1.44)	0.92 (0.67–1.27)	0.67	1	0.98 (0.71–1.35)	0.99 (0.65–1.49)	0.92 (0.52–1.63)	0.98
Model ɪɪ	1	0.98 (0.73–1.30)	0.84 (0.62–1.13)	1.02 (0.77–1.36)	0.59	1	1.04 (0.73–1.47)	1.16 (0.74–1.80)	1.11 (0.60–2.03)	0.90
Central obesity										
Crude	1	1.04 (0.85–1.26)	1.07 (0.88–1.30)	1.14 (0.94–1.39)	0.57	1	0.97 (0.80–1.18)	0.98 (0.81–1.19)	1.02 (0.83–1.24)	0.97
Model ɪ	1	1.02 (0.84–1.24)	1.07 (0.87–1.30)	1.14 (0.93–1.39)	0.57	1	0.95 (0.75–1.20)	0.96 (0.71–1.30)	1.02 (0.67–1.55)	0.90
Model ɪɪ	1	1.006 (0.74–1.36)	0.11 (0.82–1.52)	1.23 (0.90–1.66)	0.49	1	0.99 (0.69–1.42)	1.19 (0.75–1.89)	1.19 (0.63–2.25)	0.74
Hypertension										
Crude	1	0.88 (0.66–1.17)	1.01 (0.76–1.34)	0.97 (0.73–1.28)	0.77	1	1.09 (0.83–1.44)	0.87 (0.65–1.17)	1.21 (0.91–1.60)	0.17
Model ɪ	1	0.86 (0.65–1.16)	0.99 (0.74–1.32)	0.98 (0.74–1.31)	0.75	1	1.25 (0.88–1.76)	1.05 (0.67–1.65)	1.65 (0.90 3.03)	0.10
Model ɪɪ	1	0.86 (0.64–1.15)	0.99 (0.74–1.33)	0.99 (0.74–1.32)	0.71	1	1.28 (0.91–1.82)	1.12 (0.71–1.76)	1.78 (0.97–3.29)	0.08
Low HDL-C										
Crude	1	1.14 (0.94–1.39)	1.09 (0.90–1.33)	1.23 (1.01–1.49)	0.20	1	0.97 (0.80–1.17)	1.05 (0.87–1.28)	0.98 (0.81–1.20)	0.87
Model ɪ	1	1.14 (0.94–1.38)	1.10 (0.90–1.34)	1.21 (1.001–1.48)	0.24	1	0.99 (0.78–1.25)	1.09 (0.81–1.47)	1.04 (0.69–1.58)	0.85
Model ɪɪ	1	1.14 (0.93–1.38)	1.09 (0.90–1.34)	1.22 (1.001, 1.48)	0.25	1	0.99 (0.78–1.25)	1.12 (0.82–1.51)	1.07 (0.71–1.62)	0.77
Hyperglycemia										
Crude	1	1.13 (0.80–1.60)	1.19 (0.79–1.79)	1.17 (0.78–1.77)	0.38	1	0.88 (0.62–1.24)	0.71 (0.49–1.04)	0.88 (0.61–1.26)	0.39
Model ɪ	1	1.13 (0.79–1.61)	0.82 (0.56–1.21)	0.87 (0.59–1.27)	0.35	1	0.96 (0.62–1.48)	0.81 (0.46–1.44)	1.08 (0.50–2.35)	0.58
Model ɪɪ	1	1.12 (0.79–1.61)	0.83 (0.57–1.22)	0.87 (0.60–1.28)	0.39	1	0.99 (0.64–1.53)	0.86 (0.48–1.53)	1.16 (0.63–2.54)	0.60
Hypertriglyceridemia										
Crude	1	1.01 (0.80–1.28)	1.06 (0.84–1.34)	1.01 (0.80–1.28)	0.96	1	1.27 (1.01–1.59)	1.29 (1.02–1.63)	1.08 (0.85–1.38)	0.08
Model ɪ	1	1.007 (0.79–1.27)	1.04 (0.82–1.32)	1.01 (0.79–1.28)	0.98	1	1.29 (0.97–1.70)	1.35 (0.94–1.94)	1.17 (0.71–1.93)	0.11
Model ɪɪ	1	1.002 (0.79–1.27)	0.94 (0.74–1.18)	0.90 (0.71–1.14)	0.79	1	1.32 (0.99–1.75)	1.43 (0.99–2.06)	1.24 (0.75–2.06)	0.08

*Note:* Data are shown as odds ratios and 95% confidence intervals. MetS and its components were defined according to the International Diabetes Federation (IDF) and American Heart Association (AHA)/National Heart, Lung, and Blood Institute (NHLBI) criteria.

Abbreviations: DII, dietary insulin index; DIL, dietary insulin load; FBS, fasting blood sugar; HDL-C, high-density lipoprotein cholesterol; LDL-C, low-density lipoprotein cholesterol.

^a^Obtained from binary logistic regression.

^b^Adjusted for age, education, marital status, socioeconomic status, home ownership, smoking, energy intake, and physical activity.

^c^Additionally adjusted for BMI.

^d^Central obesity was defined as waist circumference ≥ 95 cm in men and women.

^e^Hypertension was defined as systolic blood pressure ≥ 130 mm Hg and/or diastolic blood pressure ≥ 85 mm Hg.

^f^Low HDL-C was defined as HDL‐C < 50 mg/dL in women and < 40 mg/dL in men.

^g^Hyperglycemia was defined as FBG ≥ 100 mg/dL.

^h^Hypertriglyceridemia was defined as triglycerides ≥ 150 mg/dL.

## Data Availability

The datasets used in this study will be available from the corresponding authors on reasonable request.

## Nomenclature

AHA: American Heart Association
BMI: Body mass index
CIs: Confidence intervals
DBP: Diastolic blood pressure
DII: Dietary insulin index
DIL: Dietary insulin load
FBG: Fasting blood glucose
FFQ: Food frequency questionnaire
FII: Food insulin index
FIL: Food insulin load
HDL: High-density lipoprotein
IDF: International Diabetes Federation
IGF-1: Insulin growth factor-1
IPAQ: International physical activity questionnaire
MCA: Multiple correspondence analysis
MetS: Metabolic syndrome
NHLBI: National Heart, Lung, and Blood Institute
ORs: Odds ratios
SBP: Systolic blood pressure
TG: Triglyceride
USDA: US Department of Agriculture
WC: Waist circumference
WSI: Wealth score index
